# What drives smallholder farmers’ willingness to pay for a new farm technology? Evidence from an experimental auction in Kenya

**DOI:** 10.1016/j.foodpol.2019.03.005

**Published:** 2019-05

**Authors:** Hira Channa, Amy Z. Chen, Patricia Pina, Jacob Ricker-Gilbert, Daniel Stein

**Affiliations:** a Purdue University, 403 W State St, West Lafayette, IN 47907, United States; b IDinsight, 785 Market Street, Suite 200, San Francisco, CA 94103, United States

**Keywords:** Hermetic storage, Experimental auction, Kenya, Medium of information

## Abstract

We use an incentive compatible experimental auction to measure demand for a new agricultural technology, a triple layered hermetic storage bag. When used properly, the bag creates an airtight seal that reduces storage loss from insect pests and neutralizes aflatoxin contamination in stored grain. We find that demand for this new technology is highly elastic (4.3) and that the wholesaler could increase profit by lowering the price. We also find that farmers’ valuation for the bag is not significantly different based on the medium through which information about it is communicated to them, either text, audio or video messages. This suggests that practitioners should use the cheapest option for disseminating information, which is text messaging in this context. In addition, we find that farmers who have prior awareness of the bag are willing to pay 20% more on average than those previously unaware of it. In total, the highly elastic demand for the improved bags, along with the fact that prior awareness of the bag leads to higher willingness to pay, suggests that a one-time price subsidy for the new technology could spur demand and increase future adoption.

## Highlights

• Demand among smallholder farmers in Kenya for improved storage bags is elastic.
• Lowering the price of the bag by 20% leads to a 29% increase in profit.
• Prior awareness of the technology increases mean willingness to pay by 20%.
• Medium by which information is disseminated does not affect willingness to pay.
• Cheapest media option, text message, is most cost-effective.

## 1 Introduction

What drives the adoption of new farm technologies amongst smallholder farmers in the developing world? This is an important question for policymakers and researchers, because new technologies can increase agriculture productivity, improve food security and help enhance the economic status of farming households. Better understanding of farmer characteristics and policy mechanisms that drive technology adoption help practitioners create programs that target those most likely to adopt a technology and benefit from using it.

Although the literature on agricultural technology adoption is extensive, very few studies have estimated demand for technologies using experimental auctions. Experimental auctions allow precise measurement of willingness-to-pay (WTP), using real stakes and products. The objective of the present article is to measure demand for a new agricultural technology, a triple layered hermetic (airtight) storage bag amongst smallholders in Kenya using an experimental BDM auction (following Becker et al., 1964). When used properly, the bag, called a Purdue Improved Crop Storage (PICS) bag, creates an airtight seal that reduces storage loss from insect pests and neutralizes aflatoxin contamination in stored grain. While the PICs bag is effective at reducing storage losses, it is significantly more expensive than traditional woven bags that offer no protection from insects or aflatoxin {roughly KSh 250 for a 90 kg PICS bag vs. KSh 80 for a 90 kg woven bag}. Therefore, adoption may not be automatic amongst limited resource smallholder farmers and they may be sensitive to price.

With this in mind, we answer three research questions related to the adoption and willingness to pay for this new storage technology: (1) How elastic is demand for the new bag? (2) Does prior awareness of the bag affect willingness to pay? (3) Is the average WTP affected by the information medium (i.e. by video, text or audio) by which farmers learn about the technology?

We answer these questions by conducting an experiment in which we randomize the medium of information through which the participant learns about the bag, and then subsequently measure their WTP using a BDM auction. This allows us to clearly measure the impact of the information medium on farmer valuations. Almost none of the 682 smallholder farmers in our sample from western Kenya had ever used the PICS bags before our auction, though some had heard about it.

Our article makes an empirical contribution to the vast literature on technology adoption, using a PICS bag as an example. Our work most closely fits in with previous studies on how farmer characteristics and behavior affect agricultural technology adoption amongst smallholders in the developing world. Work by Feder et al. (1985) point out that adoption rates are heterogeneous across farmer characteristics with risk preferences, education and tenancy status all playing a role. Recent work by Suri (2011) confirms the role of heterogeneity in returns on technology adoption among smallholders in Kenya. Fuglie and Kascak (2001) highlight the role of land quality and farm size, while Cunguara and Darnhofer (2011) find that market access can affect returns to technologies and thereby affect adoption rates. There is also growing literature documenting the role of one’s own experience, social networks and learning (Besley and Case, 1993, Foster and Rosenzweig, 1995, Conley and Udry, 2010) as possible determinants of the factors that drive adoption amongst farmers.

Our article specifically builds upon existing literature related to farmer WTP for new technologies, most of which uses stated preference methods. For example, Bell et al. (2014) use choice experiments to make the case that farmers in Pakistan will pay for irrigation services. Qaim and De Janvry, 2003, Horna et al., 2007 also use choice experiments to estimate demand for seed variety traits, while Hill et al. (2013) estimate demand for an insurance product. The challenge surrounding stated preferences methods is the inherent hypothetical bias due to the lack of actual transactions.^1^ Another problem with stated preference methods is that the difference between actual and hypothetical bids is very context specific (List and Shogren, 1998).

Recognizing this issue, the present article is among a relatively small group of papers that use experimental auctions to measure demand for agricultural products in the developing world. Stein and Tobacman (2016) measure demand for an innovative agriculture insurance product in a lab setting using BDM amongst Indian farmers. Cole et al. (2016) measure valuations for a new agriculture insurance product and an information service using two methods, BDM and the fixed price method. One of their main findings is that valuations elicited using the two different methods are largely similar. Lybbert et al. (2018) measure WTP for laser land levelling services in India to determine what type of discounts would be the most cost-effective. Waldman et al. (2014) use a Vickrey auction to determine that demand for new crop varieties is overstated when stated preference methods are used to elicit farmer WTP.

Results from our BDM auction reveal that demand for the PICS bag is highly elastic, with an elasticity estimated at 4.3 between the price range of KSh 200 and KSh 250.^2^ This high elasticity suggests that the wholesaler for PICS bags in Kenya could increase their profit by 29% if they lower the suggested retail price from KSh 250 to KSh 200. We also find that WTP is not significantly different for people who learned about the technology through either text or video messages, compared to audio messages. Prior awareness of the bag is the most important factor correlated with willingness to pay, as farmers with previous awareness of the PICS bags have a WTP that is around 20% higher on average than those with no previous knowledge of the technology.

## 2 Background

### 2.1 The technology – PICS bag

The present article is specifically concerned with estimating WTP among smallholders for a new, improved storage technology designed to reduce losses from insects, mold and rats during on-farm storage. The PICS bag developed at Purdue University in the United States is a three-layer hermetic bag that consists of an outside layer of woven polypropylene and two inner layers of polyethylene.

Without hermetic storage or other effective technologies, quantity losses due to insects, mold and rats can be a major source of loss in the grain supply chain in the developing world. For maize specifically, insect pests alone can damage 20–30% of a stored crop after six months (Boxall, 2001). In addition to these losses, there is also depreciation in the economic value of damaged maize. In a study in Benin, Kadjo et al. (2016) use revealed preference methods to measure price discounts and find that damaged grain is discounted by 3% on average, although these price discounts for damaged maize disappear as people grow desperate in the lean season.

Government response in many countries has been to advocate the use of storage insecticides like Actelic. However, one serious drawback is that use of insecticides can be extremely dangerous for consumer health if insecticide treated maize is consumed before the latency period of around three months ends (Tefera, 2012).

There is evidence that farmers who use PICS bags use the technology in place of storage chemicals. Omotilewaet al., 2018, Omotilewaet al., 2018 find in Uganda using experimental data that giving the PICS bags to farmers reduces the likelihood of using storage chemicals by 4%, and increases the length of storage. In addition, the airtight seal of the PICS bag stops mold growth and prevents the spread of aflatoxin in stored grain that is properly dried (Williams et al., 2014). Using a RCT that involved nearly 2000 smallholder households in southern Senegal, Prieto et al. (2017) compare various post-harvest technologies to find that the PICS bag is the most effective at reducing aflatoxin levels in stored maize.

PICS bags were initially disseminated on a large scale in West and Central Africa and investments were made to develop commercial supply chains of the bags. By 2014, nearly 2.5 million bags had been sold in the regions, with continued demand for more (Murdock and Baoua, 2014). As a more specific example, PICS bags were introduced in 2015 to Kenya, and a recent study in Kakamega district of western Kenya found that after just two calendar years 6% of the sample had purchased a hermetic bag (Channa and Ricker-Gilbert, 2017). It should be noted that the PICS bag can be utilized for multiple grain and legume crops.

As mentioned in the introduction, the potential drawback relative to the single layer woven bag is the PICS bag’s higher upfront cost {KSh 250 per one 90 kg bag, vs. KSh 80 for a single layer woven 90 kg bag that offers no protection against insects, molds or other pests}. However, research indicates that hermetic bags are more cost effective than alternate storage methods in the longer run. For example, Ndegwa et al. (2016) find using randomized control trial (RCT) data from Kenya that the bags are profitable if used for four seasons.^3^

## 3 Experimental design

Our experiment took place in the Western and Rift Valley province (older administrative divisions) of Kenya. The areas where the survey was conducted are major maize producers in Kenya (ICPAC Geoportal, 2017), and have two major maize seasons. The long rain season starts with planting in March-April and ends with the harvest in August-September. The short rain season (where a much smaller proportion of farmers plant maize) starts with planting in October-November and ends with the harvest in March-April. The original sample consisted of 723 farmers in our sample from six counties: Trans Nzoia, Uasin Gishu, Bungoma, Elgeyo Marakwet and Nandi. Out of this sample, 682 farmers agreed to participate in the study and provided their willingness to pay for PICS bags.^4^ The sample of farmers consisted of customers from a local microfinance bank, most of whom were taking part in a separate evaluation of an agricultural information service.^5^

Farmers were not paid a participation fee, so any purchase of PICS bags came from their own money. There is a trade-off with regards to participation fees: providing farmers money to participate eases any temporary liquidity constraints that may lessen WTP, but also might increase WTP by heightening social desirability bias. In this work we address farmer liquidity concerns at the time of the auction by allowing farmers to pay later when they could raise the money for the bag (within the next week).

Researchers from IDinsight, a non-profit research organization, managed the implementation of the auction. Prior to eliciting their WTP, farmers were presented information on the hermetic storage bags using a randomly assigned medium. One of these was a text message, the other an audio message and the third a video message (all of the messages were in Swahili).^6^ Each message was delivered to the respondent by the enumerator conducting the auction on an Android smartphone before the respondents were asked to place their bids. The content of the message across the different mediums was kept very similar. The key point of the message in each medium was that the PICS bag allowed for storage without chemicals.

After this, the participants were given an outline of the auction process and were told that it would be in their best interest to bid their maximum true valuation for the bag. Participants were also told that the bags were available in the nearby markets at prices starting from KSh 250.^7^ The participants had a practice round with biscuits, which followed the same steps as the final auction with the bag.

Briefly, the BDM auction was implemented as follows: After the practice round the participants were told to bid for the bag in multiples of ten shillings. After the bid, the enumerators explained to the participant exactly what would happen in different scenarios when the offer price was drawn, and gave them a chance to adjust their bid. For example, if the participant bid 50 shillings, the enumerators told them that if the random price that was drawn was higher, such as 60 shillings, then they would not be able to purchase the bag. If however the price drawn was lower for example 40 shillings, then they would get to purchase the bag at 40 shillings. This was repeated until the participant settled on a final bid. After the bid, the enumerator presented the participant with a bag full of sealed envelopes, and the participant chose one envelope from the bag. The participant then opened the envelope and read the price, and then the enumerator instructed the participant on the outcome of the game, which resulted in no sale or the participant purchasing the PICS bag.

If the participants did not have the required money to purchase the bag at the time of the experiment, they could arrange a time to meet with the research team in the next two weeks in order to make the purchase. The vast majority (56/58) of farmers who purchased bags did so using the mobile money service M-PESA.

## 4 Empirical model

### 4.1 Elasticity estimation

The first objective of our study is to estimate the elasticity of demand for improved storage bags. To do so, we estimate the market demand curve for the PICS bags using the WTP data from respondents in our sample. We estimate the elasticity for the bags as the proportion of individuals willing to purchase at each price using survival analysis.^8^ Eq. (1) shows the formula we used to calculate this proportion.^9^

(1) $$S_{j}=\prod_{k=1}^{j}\frac{n_{k}-d_{k}}{n_{k}}$$

In the equation above $n_{k}$ denotes the number still willing to purchase at price point k, and $d_{k}$ denotes those whose WTP was less than k. The standard errors for the proportions calculated in Eq. (1) are estimated using Eq. (2).

(2) $${\mathrm{st}}_{j}=S_{j}*\sqrt{\sum_{k=1}^{j}\frac{d_{k}}{n_{k}\left(n_{k}-d_{k}\right)}}$$

It is a simple extension to calculate the elasticity using the proportions estimated in Eq. (1) with Eq. (3) below.

(3) $$\varepsilon_{s}=\%\Delta S_{j}/\%\Delta {\mathrm{Price}}_{j}$$

The standard error for the elasticity estimate using the delta method estimated in Eq. (4) is as follows:

(4) $${\varepsilon_{\mathrm{st}}}_{s}={\mathrm{st}}_{j}*{\left(1/\Delta {\mathrm{Price}}_{j}\right)}^{2}$$

**Fig. 1:**
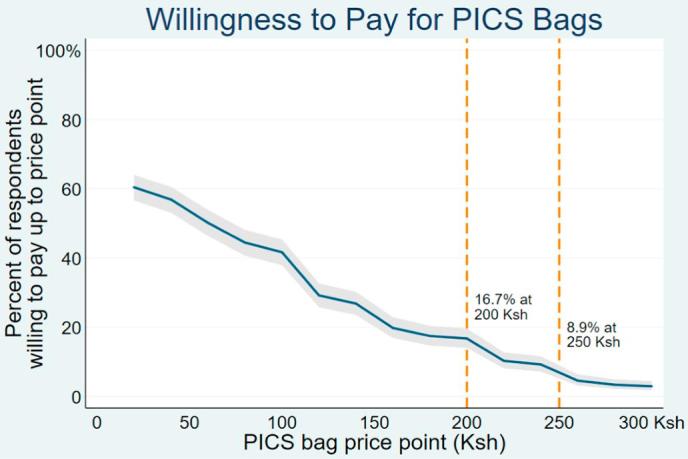
Willingness to pay for bags. The graph above is built using survival analysis. N = 682. These estimates represent the proportion of farmers willing to pay at or above a given price. Gray shaded region represents 95% confidence interval. 41 survey respondents not included in these WTP estimates because of various reasons including 6 cases where the enumerator explained the activity incorrectly. Orange lines mark out the proportion of individuals willing to buy at KSh 200 and KSH 250. (For interpretation of the references to colour in this figure legend, the reader is referred to the web version of this article.)

The proportion of individuals willing to purchase at each price is plotted against the price in Fig. 1, and the standard errors from Eq. (2) are then used to calculate the confidence intervals, also shown in Fig. 1.

### 4.2 Estimation of the determinants of WTP

The second objective of the article is to test which factors affect an individual’s WTP for the improved storage bags. We do so by estimating a model of demand for PICS bags by individual *i* as follows:

(5) $${\mathrm{WTP}}_{i}=M_{i}\gamma +A_{i}\delta +X_{i}\beta +\varepsilon_{i},\varepsilon_{i}∼N\left(0,\sigma^{2}\right)$$

WTP is the amount the respondent is willing to pay for one bag (between 0 and 300 KSh). The vector of dummy variables M, indicates the medium in which the respondent learned about the PICS bags; either through text, audio, or voice. The coefficient estimate of $\hat{\gamma }$ tests the hypothesis about whether WTP for PICS bags differs based on the medium in which information is presented to the respondent. Similarly, A is an indicator variable for whether the farmer had any prior awareness about the PICS bags before being approached for the auction, and the coefficient $\hat{\delta }$ tests the hypothesis about whether prior awareness of the technology affects WTP for it. We also include other variables that could affect farmer WTP in the vector X. This vector of variables includes respondent gender, size of farming area, quantity of maize harvested in the previous long rain season, length of period in months for which the maize was stored following the previous season and a dummy for whether the participant won in the practice round. The error term in Eq. (5) is denoted by ε. Given the experimental nature of our auction, we assume that ε is i.i.d (independent and identically distributed) normal.

### 4.3 Estimator choice

Nearly 38% of the observations in our sample have a WTP of zero, suggesting that the dependent variable exhibits properties of a corner solution variable (Wooldridge, 2010). This suggests that a linear specification estimated via Ordinary Least Squares (OLS) is likely to be biased.

The tobit specification provides an opportunity to deal with the corner solution nature of our dependent variable, WTP. However, a concern with the tobit is that it assumes the same underlying process for those who bid zero, and those who bid values greater than zero.

Fortunately, hurdle models are more flexible because they separate out the underlying decision into two. The first step involves the decision to “participate”; in our case this would be the decision on whether to bid for the bag at all. The next step is then the decision of how much to pay. We use the Cragg (1971) hurdle model which he specified to explain demand for durable goods.

(6) $$s_{i}=\left\{\begin{array}{cc} 1 & ifM_{i}\gamma +A_{i}\delta +X_{i}\beta +\varepsilon_{i}>0\\ 0 & \mathrm{otherwise} \end{array}\right)$$

The continuous variable ${\mathrm{WTP}}_{i}$ is observed only if $s_{i}=1$, and is modeled as in Cragg (1971):

(7) $${\mathrm{WTP}}_{i}=M_{i}\gamma +A_{i}\delta +X_{i}\beta +v_{i}$$

In the specification above $v_{i}$ has a truncated normal distribution, where it is truncated at $M_{i}\gamma_{v}+A_{i}\delta_{v}+X_{i}\beta_{v}$.

## 5 Results and discussion

### 5.1 Data description

Table 1 presents the key descriptive statistics for our sample of 682 respondents. We present the means and standard deviations for six key variables for all our respondents and by information medium (audio, text, and video) through which they received information about PICS bags.^10^ The average WTP for the entire sample is KSh 83. A little more than half of the sample is female and the average farm size is 2 acres. Almost all the respondents (87%) stored some grain in the previous season, and the average maize harvested was 2717 kg for the previous season.^11^ Additionally 38% of the respondents had a final bid of zero for the bag. We check for balance amongst the different media groups by using a multinomial logit regression, following Mckenzie (2015). Results suggest that we are unable to reject the null that these characteristics are the same for households across the different categories (information types) at a p-value of 0.51.

**Table 1:** Summary statistics by media type.

	Audio	Text	Video	All
PICS WTP bid for analysis (KSh)	81	90	81	83
	(92)	(90)	(81)	(92)
Prior PICS awareness	0.27	0.24	0.19	0.23
	(0.45)	(0.42)	(0.39)	(0.42)
Respondent is female (binary)1 = Female	0.56	0.54	0.56	0.55
	(0.50)	(0.50)	(0.50)	(0.50)
Total Maize Harvested(metric tons)	2.70	2.40	2.50	2.50
	(4.50)	(2.90)	(4.70)	(4.10)
Farm Size(Acres)	1.95	2.00	1.90	2.00
	(2.10)	(2.30)	(2.30)	(2.30)
Months the maize was left in storage during the previous season(months)	1.60	1.60	1.60	1.60
	(1.50)	(1.80)	(1.60)	(1.60)
Individual won biscuit in demonstration round (Won = 1)	0.58	0.56	0.62	0.59
	(0.49)	(0.5)	(0.49)	(0.49)
Observations	229	194	241	664

Standard deviations in parentheses; Notes-In order to check for balance across categories we run a multinomial logit using the media type as the dependent variable. See Mckenzie (2015) (http://blogs.worldbank.org/impactevaluations/tools-trade-joint-test-orthogonality-when-testing-balance). The p- value for a joint hypothesis test is 0.505 indicating that we cannot reject the null hypothesis that that the means of each of these variable is not statistically different across the different groups.

Table 1 includes statistics from 664 observations, for which complete data was available for all variables.

### 5.2 Demand and profitability analysis

Fig. 1 presents the demand curve for PICS bags from our sample, which is based on the proportions estimated from the survival analysis at each price point. We use Eqs. (3), (4) discussed above to measure elasticity and the associated standard error. The price elasticity between the prices of KSh 200 and KSh 250 (the current retail price, and the suggested retail price based on the profitability calculation described below) is 4.3 [0.81]^12^ suggesting highly elastic demand. As the price falls by KSh 50 demand increases from 8.9% to 16.7% of the sample who are willing to purchase the bag (Fig. 1).

Based on the demand curve, we now consider whether the wholesaler of the bags could increase profit by lowering the wholesale and through this the retail price of the bags.^13^ This analysis focuses on the retailer’s and wholesaler’s profit, and assumes that the ratio of the wholesale price to the retail price remains constant no matter what price the wholesaler sets.

**Fig. 2:**
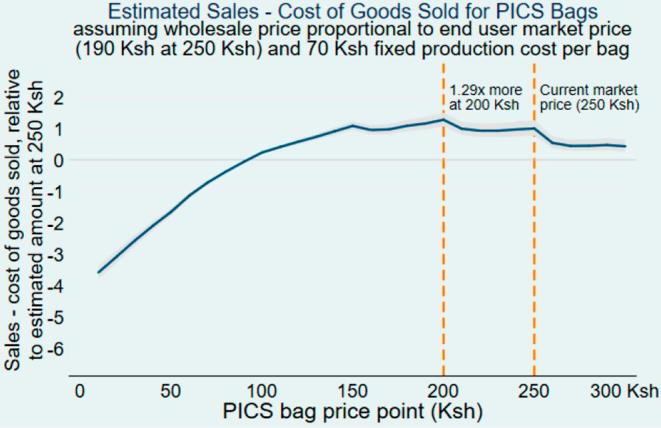
Profitability analysis. N = 682. These estimates are calculated as the sales – cost of goods sold per bag (price point * 190 Ksh (Bell wholesale price)/250 Ksh (end user market price) − 70 Ksh (fixed production cost per bag)) multiplied by the percentage of respondents willing to pay at that price. The estimates are then divided by the estimated profit at 250 Ksh. This model does not incorporate varying production costs by volume, nor other marketing and distribution costs. This model does not incorporate varying production costs (input and manufacturing costs) by volume, nor other marketing and distribution costs. Gray shaded region represents 95% confidence interval.

At the time of the auction the wholesale price for retailers to purchase one PICS bag was KSh 190, equivalent to 76% of the current suggested retail price of KSh 250. We assume that the ratio of the wholesale to retail price always stays the same at 76%. This simplified analysis also ignores potential economies of scale in production costs, and assumes a fixed production cost of KSh 70 per bag. We assume KSh 70 to be the cost per bag to the wholesaler, since in our scenario the wholesaler and manufacturer are one entity. Fig. 2 provides the profit of the wholesaler under these assumptions at price points ranging from KSh 0 to KSh 300. The profit is scaled by dividing it with the profit of the wholesaler at the current price of KSh 250. It turns out that the profit (in this simplified scenario) is highest when the retail price is KSh 200, and the wholesale price is KSh 152, 76% of KSh 200.

If the retail price is lowered from KSh 250 to KSh 200 (by lowering the wholesale price KSh 190 to KSh 152), the price decrease of 20% is accompanied by an increase in demand from 8.9% to 16.7%. For the wholesaler this works out to a 51% increase in revenue and 1.29 times more profit (Fig. 2).

In order to further illustrate this point we develop a scenario imagining that we have a total potential market (number of farmers who could buy PICS bags) of 1000. We use this scenario to show how sales and profit, of the retailer and the wholesaler, would change if the retail price is lowered from KSh 250 to KSh 200. In the status quo, the suggested retail price for one PICS bag is KSh 250, and the wholesale price is KSh 190. Our demand analysis indicates that 9% of the potential market purchases the bag at a retail price of 250, so 90 bags are sold. With 90 bags sold, the retailer’s revenue is KSh 22,500 {KSh 250 * 90 bags} and her profit is KSh 5400 {(KSh 250–190) * 90 bags}. In this situation the wholesaler’s revenue is KSh 17,100 {KSh 190 * 90 bags} and her profit is KSh 10,800 {(KSh 190–70) * 90 bags}.

Now we look at what happens when the retail price of PICS bags is lowered to KSh 200, and the wholesale price to KSh 152. Demand would now go up to approximately 170 bags sold. Now the retailer’s revenue is KSh 34,000 {KSh 200 * 170 bags} and her profit is KSh 8160 {(KSh 200–152) * 170 bags}. The wholesaler’s revenue is KSh 25,840 {KSh 152 * 170 bags} and her profit is KSh 13,940 {(Ksh 152–70) * 170 bags}.

This example shows us that by lowering the retail and wholesale price of PICS bags by 20% demand goes up from 8.9% to 16.7%, the retailer profit goes up by 51% {(8160–5400)/5400} and the wholesaler profit goes up by 29% {(13,940–10,800)/10,800}.

One limitation should be noted with regards to our demand and profit calculations. The result from the BDM auction cannot be extrapolated to a scenario where participants are purchasing multiple bags, because we elicit willingness to pay for only the first bag. It is possible that the willingness to pay for any bags thereafter is different. This is especially the case given the fact that the average farmer needs more than one 90 kg bag to store all of his or her maize.

### 5.3 Factors affecting willingness to pay for hermetic bags

Table 2^14^ presents results from multiple empirical specifications of factors that affect WTP for hermetic bags. The first two columns present results from a parsimonious specification of a linear model estimated via OLS, while the next column is a full specification using OLS. The next two columns present the result from the tobit and Cragg’s hurdle model respectively. All specifications give similar results.

**Table 2:** Factors affecting willingness to pay for PICS bags (in Kenya shillings).

	(1)	(2)	(3)	(4)	(5)
	OLS (parsimonious specifications)		OLS (Full Model)	Tobit	Hurdle Model
Prior PICS awareness (binary)1 = Aware	20.95^*^*		15.93**	20.19*	15.12**
	(8.89)		(7.864)	(11.99)	(7.420)
Respondent is female (binary)1 = Female			−0.926	2.159	−0.122
			(6.747)	(10.37)	(6.686)
Total Maize Harvested (metric tons)			−0.000281	−0.000221	−8.32e-05
			(0.00159)	(0.00242)	(0.00173)
Farm Size (Acres)			3.244	4.076	2.882
			(2.706)	(4.122)	(2.483)
Shown a text message explaining the technology¥		8.88	10.57	18.21	10.27
		(9.24)	(8.355)	(12.87)	(8.408)
Shown a video explaining the technology¥		−0.095	−1.255	3.544	−0.317
		(8.39)	(7.900)	(12.19)	(7.674)
Months the maize was left in storage during the			1.809	4.600	1.814
previous season (months)			(2.045)	(3.145)	(2.055)
Individual won biscuit in demonstration round			70.77***	132.9***	60.70***
(Won = 1)			(6.741)	(11.11)	(6.155)
R-squared	0.013	0.005	0.024	0.0025	0.0039

Robust standard errors in parentheses; OLS results and tobit and double hurdle marginal effects reported; Dummies for two areas of Eldoret and Kitale are included in the specification.

¥ Compared to a control of audio message; Total number of observations in each specification is 664; ^***^p < 0.01, ^**^p < 0.05, ^*^p < 0.1; McFadden’s pseudo R-squared reported for the tobit and Craggs hurdle regression.

Our results suggest that regardless of estimator and/or specification, the WTP for PICS bags is not different between text message, video message and audio message. The finding that the different mediums for marketing messages are not associated with different WTP has practical implications for policymakers and businesses looking to inform farmers about new technology. Given that the WTP is not statistically different across communication mediums, the focus should be on the medium which is least expensive in terms of reaching the most individuals per dollar or Shilling spent. For example, within the context of this study, it costs KSh 31,250 to send two text messages to 5000 farmers per week, equivalent to KSh 3.13 per text message. It costs KSh 72,500 to send two audio messages to 5000 farmers per week, equivalent to KSh 7.25 per audio message. Sending video messages is much more expensive and it would cost approximately KSh 105,000 to send just one video message to 5000 farmers, equivalent to KSh 21 per video message.^15^

Our findings predict that text messages have the same impact on demand, while being considerably cheaper than marketing based on audio or video messages. Another factor that might affect the medium used is the literacy level of the households that are being targeted as potential customers. In our case this does not appear to be a major concern as 90% of the respondents in our survey stated that they were able to read the text message, so text message appears to be a cheap (in terms of KSh spent on reaching a respondent) and accessible medium in this context. However if scaling up to a less literate population, then audio messages might be the most suitable medium to reach the most people.

Results from Table 2 also indicate that across specifications, having previous awareness of hermetic bags increases the average respondent’s WTP for PICS bags by KSh 15.12–20.95 compared to those who are unaware. This result is robust and statistically significant (p-value < 0.05) across the different specifications, and corresponds to a relative increase in mean WTP of around 20%.

We also find that the dummy for winning in the practice round is highly significant. This is an interesting find, and it seems likely that this is significant in the model is because WTP for biscuits and PICS bags is positively correlated. If an individual is willing to pay more for one, he or she is also willing to pay more for the other. Also naturally, in a BDM auction, if you bid more you are more likely to win.

None of the coefficients on additional individual characteristics that we include in the regression, (i.e. gender, farm size, months maize was in storage and quantity of maize harvested), are significantly different from zero. While there may be other factors affecting the variation in WTP that we do not observe in our model, our experimental design should control for concerns about biased coefficient estimates. Recall from Table 1 that the medium of information shown to a particular farmer (audio, video, text) was randomly chosen, so demographic variables are balanced across medium of information as shown in Table 1 which we discussed earlier.

## 6 Conclusions and policy implications

The present article uses a Becker-DeGroote-Marshack (BDM) auction to estimate willingness to pay (WTP) for a new farm technology (PICS hermetic storage bags), amongst a sample of 682 smallholder maize farmers in western Kenya. The hermetic storage technologies are more effective than traditional woven bags at eliminating insect, mold and other pests during storage, but are significantly more expensive than other bags available at the market. Awareness was low and adoption was non-existent among our sample during the time of the auction, making this a useful case study on WTP and adoption for researchers and practitioners working towards improving farm technology adoption in the developing world.

We find that demand for the hermetic bags in our sample is highly elastic (elasticity is 4.3). High elasticity of demand for the bags is understandable in this context as people may be unsure of the technology’s benefits relative to its price. A simple analysis of profit suggests that the manufacturer of PICS bags in Kenya can increase their profits by lowering wholesale prices. For example, lowering the bag’s price by 20% from KSh 250 to KSh 200 would increase profit for the retailer by 51% and profits for the wholesaler by 29%.

Another key finding is that the medium of information about the technology (text, video or voice) does not affect willingness to pay. This finding holds significant practical value. If there is no differential impact on willingness to pay for the technology between all mediums, then practitioners should use the most cost-effective method to spread awareness about new technologies. While text-messages are clearly the most cost-effective method in our context, audio and video messaging could be more appropriate in different places depending on relative costs of using text, vs. audio, vs. video messaging and the literacy of the target population.

While most of the observed individual characteristics do not affect farmer valuation, the one exception is that prior awareness is a statistically and economically significant factor positively correlated with willingness to pay for the bag. This finding is closely linked with the results from a RCT in Uganda examining the impact of initial subsidies on adoption of a new hermetic storage technology (Omotilewaet al., 2018, Omotilewaet al., 2018). Their results (based on the same hermetic technology described in this paper) suggest that when there is uncertainty surrounding a new technology, a one-time subsidy can raise demand. This is a useful lesson for those who are looking to introduce new technologies to farmers, suggesting that initial (and temporary) subsidies for a new technology can be an effective way of scaling up adoption.

In addition, it should be noted that awareness and use of hermetic technology among our sample is low. Recall from Table 1 that only 23% of respondents are aware of hermetic technology, while only three respondents in the sample actually use hermetic technology (0.04%). It is possible that once these respondents become more aware and have experience using hermetic bags their WTP will increase, and the profit maximizing price for the bag for manufacturers and retailers may also increase. This would suggest that some investment in extension and advertising by actors in the PICS supply chain to raise awareness about the technology could be profitable for them.

Declaration of interest

None.

## Funding

IDinsight has partnered with PICS and Bell Industries to conduct a willingness to pay exercise with farmers surrounding Kitale and Eldoret to determine smallholder farmers’ willingness to pay for PICS bags in these areas, and whether demand varies by which informational message farmers receive about the PICS bag (text, audio, or video).

We would like to acknowledge the support of the Bill & Melinda Gates Foundation under the Decision-Focused Evaluations in Agriculture (DFEA) and PICS3 project, and the USAID FtF Food Processing and Post-Harvest Handling Innovation Lab.

## Figures and Tables

**Table A1:** Factors affecting willingness to pay for PICS bags using full sample (in KSh).

	OLS (Full Model)	Tobit	Hurdle Model
Prior PICS awareness (binary)1 = Aware	19.83^**^	25.47^**^	17.89^**^
	(8.485)	(12.92)	(8.116)
Respondent is female (binary)1 = Female	−1.832	−2.498	−1.431
	(7.287)	(11.15)	(7.080)
Total Maize Harvested (metric tons)	−0.000133	0.000487	9.21e−05
	(0.00172)	(0.00261)	(0.00189)
Farm Size(Acres)	3.573	3.840	2.982
	(2.86)	(2.81)	(2.78)
Shown a text message explaining the technology¥	8.998	10.67	6.231
	(9.023)	(13.77)	(8.862)
Shown a video explaining the technology¥	1.758	7.555	2.118
	(8.527)	(13.15)	(8.337)
Months the maize was left in storage during the	1.418	4.170	1.601
previous season (months)	(2.209)	(3.413)	(2.261)
R-squared	0.022	0.0025	0.0039

Robust standard errors in parentheses; OLS (parsimonious specification) results and tobit and double hurdle marginal effects reported; Dummies for two areas of Eldoret and Kitale are included in the specification.

¥ Compared to a control of audio message; Total number of observations in each specification is 682; ^***^p < 0.01, ^**^p < 0.05, ^*^p < 0.1; McFadden’s pseudo R-squared reported for the tobit and Craggs hurdle regression; This table is added as a robustness check by rerunning the specifications with means of the missing values added. The coefficients do not change qualitatively

**Table A2:** Summary statistics by bid.

	Bid greater than Zero	Bid is zero	Total
Prior PICS awareness	.25	.22	.23
	(.43)	(.41)	(.42)
Respondent is female (binary)1 = Female	.55 (.5)	.56 (.5)	.56 (.5)
Total Maize Harvested (kg)	2668 (4152)	2167 (3052)	2478 (3780)
Farm Size (Acres)	2.1	1.7	1.9
	(2.4)	(1.9)	(2.2)
Months the maize was left in storage during the previous season (months)	1.7 (1.6)	1.4 (1.6)	1.6 (1.6)

Observations	413	251	664

Standard deviations in parentheses; Table includes statistics from 664 observation, for which complete data was available for all variables

## Appendix A

See Table A1, Table A2.

## Footnotes

1 In some contexts cost and/or logistics concerns might make BDM or other revealed preference methods impossible to conduct, leaving stated preference methods as the only option.

2 Ksh 100= US $1.00.

3 To get a sense of the PICS bag usage cost versus the standard gunny when pesticide is used: Cost of 1 synthetic bag = KSh 80 + Cost of Pesticide per bag = KSh 13 + Application Labor per bag = KSh 38 = Total Cost = KSh 131/bag. This is smaller than the cost of the PICS bag at KSh 250. However, even assuming that both methods provide equivalent protection against loss, this simple back of the envelope calculation suggests that the PICS bag is more cost effective if it is used for at least two years. Anecdotal evidence suggests that it can last 3 years.

4 20 farmers were surveyed but did not enter the BDM part of the study because they withdrew consent. Frequently this was because they gave moral/ethical/religious reasons for not wanting to participate in the auction. Additionally 21 of those who had a successful bid for the bag and agreed to pay later but then did not pay. We do not consider any of these respondents so we have 682 bids out of the 723 surveyed.

5 In order to increase the sample size for this study, we recruited additional farmers were not part of the agricultural information evaluation to participate in this study. These participants were also customers of the local microfinance back, and consist of 146 of the 723 farmers in our sample. And it included 123 out of the 682 farmers from whom we got the final bid. Reported WTP for PICS bags was not significantly different between the farmers who were part of the separate evaluation and the others recruited solely for this study (p value = 0.6687).

6 The specific messages used were developed from a Bell flyer, a PICS audio jingle made for Tanzania (https://www.youtube.com/watch?v=vAgQMAKOHO8&feature=youtu.be), and a PICS video made for Tanzania.

7 The reason we decide to inform participants about the market price, is because we have heterogeneity in our sample regarding how well informed the participants are about the market. In order to control for this heterogeneity with regards to market information, everyone was informed about the current market price. Although stating a market price may anchor WTP, we believe that having some concept of an accepted price for an item provides a realistic context for people to make valuations.

8 The proportion of participants willing to pay at each price is equivalent to the quantity demanded since each participant had the option to bid for only one bag.

9 Eqs. (1), (2) have been taken from StataCorp (2014). The equations in this manual have been adapted from Kalbfleisch and Prentice (2002).

10 We also present these statistics by those presented the data by those who bid zero and those who bid above zero in Appendix Table A2.

11 We focus only on maize as our primary control and not beans which is the other main crop in this area that could be stored in PICS bags. Unfortunately, we do not have bean storage related data for 329 of our respondents. This occurred because for a random subsample the questionnaire size was reduced due to time issues. However, the remaining data suggests that this not a major issue because a majority of the remaining households, 85%, do not store any beans at al.

12 The elasticity estimate is 4.3 and 0.81 is the associated standard error.

13 In this scenario since Bell Industries manufacturers and then distributes to retailer, we assume that the manufacturer and wholesaler is one entity. In the rest of the paper we refer only to the wholesaler.

14 Table 2 provides the result for 664 observations, because of incomplete data for a few variables. As a robustness check we run the same regressions with the mean of the missing value and regress with 682 observations. The results provided in Appendix Table A1 are qualitatively the same.

15 Costs for audio and text estimated using EchoMobile’s pricing for Kenya (https://www.echomobile.org/public/platform), which includes a fixed monthly service subscription cost plus cost per message sent. Costs for video messages are based on cost of sending a MMS through Airtel. We estimate that the smallest size of the compressed vide would be 300 KB which would mean three MMS to send the video. One MMS costs KSh 7. (http://africa.airtel.com/wps/wcm/connect/africarevamp/kenya/home/business/messaging-mms).

## References

[b0005] Becker G.M., DeGroot M.H., Marschak J. (1964). Measuring utility by a single-response sequential method. Syst. Res. Behav. Sci..

[b0010] Bell A.R., Shah M., Ward P.S. (2014). Reimagining cost recovery in Pakistan's irrigation system through willingness-to-pay estimates for irrigation water from a discrete choice experiment. Water Resour. Res..

[b0015] Besley T., Case A. (1993). Modeling technology adoption in developing countries. Am. Econ. Rev..

[b0020] Boxall R.A. (2001). Post-harvest losses to insects—a world overview. Int. Biodeter. Biodegrad..

[b0025] Channa, H., Ricker-Gilbert, J., 2017.“Willingness to Pay for a new farm technology given Risk Preferences. Evidence from an experimental auction in Kenya. In: Food Processing Lab Meeting, 23 April 2017, West Lafayette, IN.

[b0030] Cole, S., Fernando, A.N., Stein, D., Tobacman, J., Business, H., Wharton, I., 2016. Field Comparisons of Incentive-Compatible Preference Elicitation Techniques.

[b0035] Conley T.G., Udry C.R. (2010). Learning about a new technology: Pineapple in Ghana. Am. Econ. Rev..

[b0040] Cragg J.G. (1971). Some statistical models for limited dependent variables with application to the demand for durable goods. Econ.: J. Econ. Soc..

[b0045] Cunguara B., Darnhofer I. (2011). Assessing the impact of improved agricultural technologies on household income in rural Mozambique. Food Policy.

[b0050] Feder G., Just R.E., Zilberman D. (1985). Adoption of agricultural innovations in developing countries: a survey. Econ. Develop. Cultural Change.

[b0055] Fuglie K.O., Kascak C.A. (2001). Adoption and diffusion of natural-resource-conserving agricultural technology. Rev. Agric. Econ..

[b0060] Foster A.D., Rosenzweig M.R. (1995). Learning by doing and learning from others: human capital and technical change in agriculture. J. Political Econ..

[b0065] Hill R.V., Hoddinott J., Kumar N. (2013). Adoption of weather-index insurance: learning from willingness to pay among a panel of households in rural Ethiopia. Agric. Econ..

[b0070] Horna J.D., Smale M., von Oppen M. (2007). Farmer willingness to pay for seed-related information: rice varieties in Nigeria and Benin. Environ. Develop. Econ..

[b0075] ICPAC GeoPortal, 2017. Kenya – Maize production statistics. Retrieved June 9 2018, http://geoportal.icpac.net/layers/geonode%3Aken_maize_production#more.

[b0085] Kadjo D., Ricker-Gilbert J., Alexander C. (2016). Estimating price discounts for low-quality maize in sub-Saharan Africa: evidence from Benin. World Develop..

[b0090] Kalbfleisch, J.D., Prentice, R.L., 2002. The survival analysis of failure time data, second ed. Hoboken.

[b0185] List J.A., Shogren J.F. (1998). Calibration of the difference between actual and hypothetical valuations in a field experiment. J. Econ. Behav. Organ..

[b0095] Lybbert T.J., Magnan N., Spielman D.J., Bhargava A.K., Gulati K. (2018). Targeting technology to increase smallholder profits and conserve resources: experimental provision of laser land-leveling services to indian farmers. Econ. Develop. Cultural Change.

[b0100] Mckenzie, D., 2015, February 4th. Tools of the Trade: a joint test of orthogonality when testing for balance [Blog post]. Retrieved from http://blogs.worldbank.org/impactevaluations/tools-trade-joint-test-orthogonality-when-testing-balance.

[b0110] Murdock L.L., Baoua I.B. (2014). On Purdue Improved Cowpea Storage (PICS) technology: background, mode of action, future prospects. J. Stored Products Res..

[b0115] Ndegwa M.K., De Groote H., Gitonga Z.M., Bruce A.Y. (2016). Effectiveness and economics of hermetic bags for maize storage: results of a randomized controlled trial in Kenya. Crop Protect..

[b0120] Omotilewa, O.J., Ricker-Gilbert, J., Ainembabazi, J.H., 2018a. Subsidies for agricultural technology adoption: Evidence from randomized experiment in Uganda. In: CSAE Conference 2018: Economic Development in Africa, Oxford, UK. 10.1093/ajae/aay108 PMC7714250 33281194

[b0125] Omotilewa O.J., Ricker-Gilbert J., Ainembabazi J.H., Shively G.E. (2018). Does improved storage technology promote modern input use and food security? Evidence from a randomized trial in Uganda. J. Develop. Econ..

[b0130] Prieto, Stacy, Bauchet, Jonathan, Ricker-Gilbert, Jacob, 2017. How do improved drying and storage practices influence aflatoxin spread? Evidence from smallholder households in Senegal. In: 2017 Annual Meeting, July 30-August 1, Chicago, Illinois. No. 258497. Agricultural and Applied Economics Association.

[b0135] Qaim M., De Janvry A. (2003). Genetically modified crops, corporate pricing strategies, and farmers' adoption: the case of Bt cotton in Argentina. Am. J. Agric. Econ..

[b0140] StataCorp, 2014. STATA survival Analysis Release Manual. Release 14. Stata Press, College Station, TX.

[b0145] Stein D., Tobacman J. (2016). Weather insurance savings accounts. Geneva Papers Risk Insurance-Issues Practice.

[b0150] Suri T. (2011). Selection and comparative advantage in technology adoption. Econometrica.

[b0155] Tefera T. (2012). Post-harvest losses in African maize in the face of increasing food shortage. Food Security.

[b0160] Waldman K.B., Kerr J.M., Isaacs K.B. (2014). Combining participatory crop trials and experimental auctions to estimate farmer preferences for improved common bean in Rwanda. Food Policy.

[b0165] Williams S.B., Baributsa D., Woloshuk C. (2014). Assessing Purdue Improved Crop Storage (PICS) bags to mitigate fungal growth and aflatoxin contamination. J. Stored Products Res..

[b0170] Wooldridge J.M. (2010). Econometric Analysis of Cross Section and Panel Data.

